# Xanthan-Gum/Pluronic-F-127-Based-Drug-Loaded Polymeric Hydrogels Synthesized by Free Radical Polymerization Technique for Management of Attention-Deficit/Hyperactivity Disorder

**DOI:** 10.3390/gels9080640

**Published:** 2023-08-08

**Authors:** Muhammad Suhail, I-Hui Chiu, Yi-Ru Lai, Arshad Khan, Noorah Saleh Al-Sowayan, Hamid Ullah, Pao-Chu Wu

**Affiliations:** 1 School of Pharmacy, Kaohsiung Medical University, 100 Shih-Chuan 1st Road, Kaohsiung 80708, Taiwan; u112830001@kmu.edu.tw (I.-H.C.); u110830701@kmu.edu.tw (H.U.); 2 Department of Pharmaceutics, Faculty of Pharmacy, Khawaja Fareed Campus (Railway Road), The Islamia University of Bahawalpur, Punjab 63100, Pakistan; arshadpharma77@gmail.com; 3 Department of Biology, College of Science, Qassim University, Buraydah 52377, Saudi Arabia; nsaoiean@qu.edu.sa; 4 Drug Development and Value Creation Research Center, Kaohsiung Medical University, Kaohsiung 80708, Taiwan

**Keywords:** attention-deficit/hyperactivity disorder, atomoxetine, porosity, swelling, drug release

## Abstract

Smart and intelligent xanthan gum/pluronic F-127 hydrogels were fabricated for the controlled delivery of atomoxetine HCl. Different parameters such as DSC, TGA, FTIR, XRD, SEM, drug loading, porosity, swelling index, drug release, and kinetics modeling were appraised for the prepared matrices of hydrogels. FTIR confirmed the successful synthesis of the hydrogel, while TGA and DSC analysis indicated that the thermal stability of the reagents was improved after the polymerization technique. SEM revealed the hard surface of the hydrogel, while XRD indicated a reduction in crystallinity of the reagents. High gel fraction was achieved with high incorporated contents of the polymers and the monomer. An increase in porosity, drug loading, swelling, and drug release was observed with the increase in the concentrations of xanthan gum and acrylic acid, whereas Pluronic F-127 showed the opposite effect. A negligible swelling index was shown at pH 1.2 and 4.6 while greater swelling was observed at pH 7.4, indicating a pH-responsive nature of the designed hydrogels. Furthermore, a higher drug release was found at pH 7.4 compared to pH 1.2 and 4.6, respectively. The first kinetics order was followed by the prepared hydrogel formulations. Thus, it is signified from the discussion that smart xanthan gum/pluronic F-127 hydrogels have the potential to control the release of the atomoxetine HCl in the colon for an extended period of time.

## 1. Introduction

Attention-deficit/hyperactivity disorder (ADHD) is one of the most common neurodevelopmental disorders in childhood and adolescence. This disorder affects between 2.2% and 17.8% of all school-aged children and adolescents. Different developmental deficits such as learning limitation, control of executive functions, as well as global impairments of social skills are associated with ADHD in children [1]. As early as 1902, “deficiencies in “volitional inhibition” and an excessive inability to pay attention for long periods of time” were seen with a group of restless children. In 1937, it was discovered that the levels of hyperactivity and behavioral issues could be reduced by amphetamine. In the 1950s, the term “minor brain damage” (MBD) was often used to describe these symptoms in children; however, in most cases, no evidence of neurological damage was identified. Until 1960, the disorder was known and labeled as ADHD and attention deficit disorder (ADD). ADHD symptoms, inattention and impulsivity (and ADHD hyperactivity), have become attributed to a syndrome. The question of whether ADHD is a separate disorder or a continuum that serves as a risk factor for future adversities is one that is still being debated. With each new diagnostic system, the symptoms have changed, and the World Health Organization diagnostic system (ICD-10) now labels it as hyperkinetic disorder and the American Medical Association System (DSM-IV) describes it as ADHD, which look similar to each other [2]. Hence, different drugs such as atomoxetine HCl (ATMH) and a number of other drugs, including certain antidepressants and -agonists used off-label, have been added to the pharmacotherapy of ADHD [3]. The different recommended psychostimulants and other medications in the treatment of children and adolescents with ADHD are Methylphenidate, Ritalin Medikinet, Concerta, and the Amphetamine liquid, respectively. Cognitive behavioral therapy, neuropsychological treatment, noninvasive brain stimulation, and other multimodal treatments are the different non-pharmacological approaches that are used for the management of ADHD [4].

ATMH is approved in the US for the management of ADHD patients [5]. Its absorption occurs very rapidly after ingestion. The bioavailability is only affected slightly by the intake of food, so the drug can be taken independently of meals. Absolute bioavailability is commonly reached 63% after oral delivery, while the maximum plasma concentration is reached 1 to 2 h after ingestion. At therapeutic concentrations, the atomoxetine–albumin binding ratio is 98% in the plasma. High pre-systemic metabolism and albumin binding occur. The half-life is 4–5 h. Cytochrome P450-2D6 isoenzyme is responsible for the degradation of ATMH in the liver [5]. The available dosage form is a capsule with different doses of 10, 18, 25, 40, 60, 80, and 100 mg for adults and pediatrics, with a dose frequency of 40 mg PO once daily at first; 80 mg PO once daily or divided every 12 h after that; and maybe 100 mg if the desired reaction is not attained. ATMH presents some problems when ingested orally. It has a very short half-life and is absorbed very rapidly. Upset stomach, nausea, vomiting, constipation, loss of appetite, dry mouth, headache, lethargy, feeling drowsy, or weakness during the day are common adverse effects, while serious side effects like liver damage, elevated suicidal thoughts, angioedema, and heart problems are associated with a single-dose administration per day. Several researchers have prepared different carrier systems for ATMH delivery. Teaima and coworkers prepared solid lipid nanoparticles for the brain targeting of ATMH and sustained the release of ATMH for 8 h [6]. Mohanty et al. (2023) prepared ATMH-loaded nanostructured lipid carriers and demonstrated the sustained release of ATMH for 12 h [7]. Similarly, Stanojevic and coworkers prepared tablets for the sustained release of ATMH of up to 8 h [8]. Yet, further research work is needed to not only sustain the release of ATMH, but also combat its adverse effects following oral delivery. Hydrogel with unique properties has been used as one of the most suitable agents for controlled drug delivery systems [9]. Khalid et al. (2018) prepared polymeric hydrogels of chondroitin sulfate for the controlled release of loxoprofen [10]. Similarly, Malik and coworkers prepared chitosan/Beta-cyclodextrin-based hydrogels for acyclovir-controlled release [11].

**Scheme 1:**
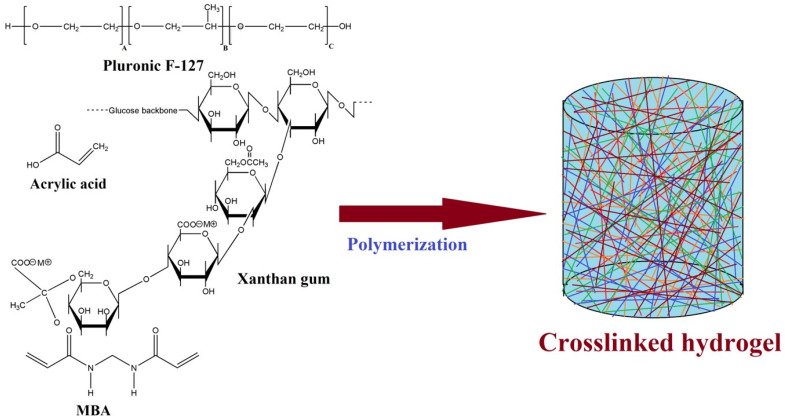
Gelation mechanism of XG/PF-127 hydrogel.

In the current study, the authors report ATMH-loaded polymeric hydrogels of xanthan gum and pluronic F-127 fabricated by the free radical polymerization technique. The combination of natural polymer xanthan gum and synthetic polymer pluronic F-127 with synthetic monomer acrylic acid increased the pH sensitivity of the fabricated hydrogels. The mechanical strength and stability of the developed hydrogels were increased due to the intermixing of natural and synthetic contents, which not only enhanced the swelling and loading of drugs, but also sustained the release of ATMH for an extended period of time (96 h). Characterization techniques such as FTIR, TGA, DSC, XRD, and SEM were performed for the formulated hydrogels. Similarly, a set of studies including sol–gel analysis, porosity, swelling, drug loading and release, along with kinetics modeling have been performed for the fabricated networks of hydrogel. Gelation mechanism of prepared hydrogels is shown below in Scheme 1.

## 2. Results and Discussion

### 2.1. FTIR Analysis

**Figure 1:**
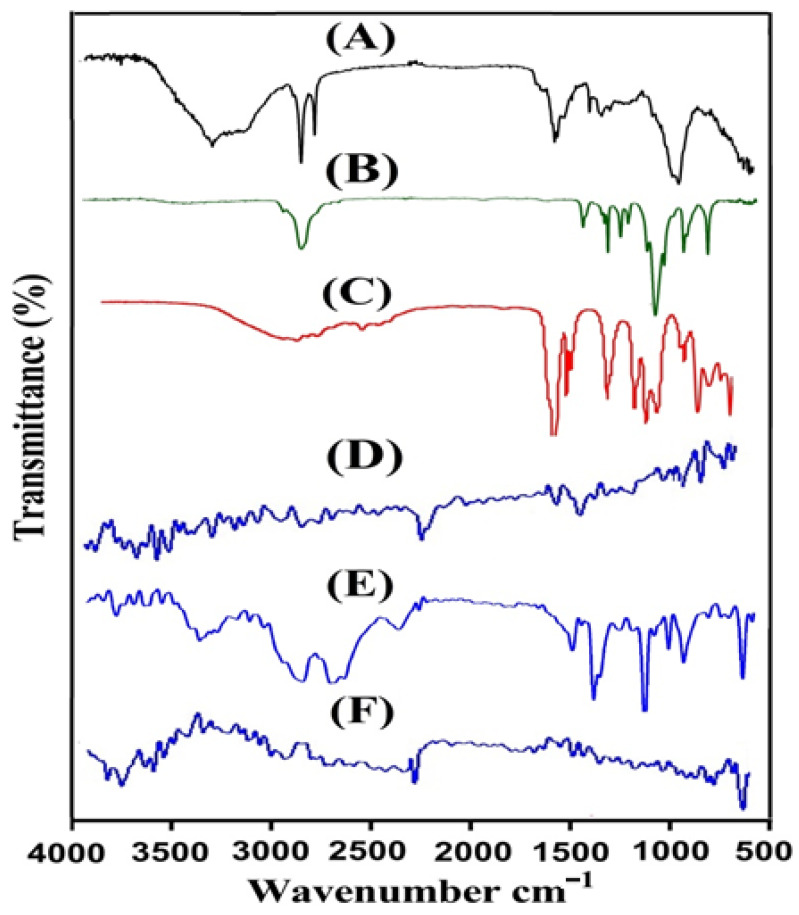
FTIR spectra of (**A**) XG, (**B**) PF-127, (**C**) Aa, (**D**) the unloaded XG/PF-127 hydrogel, (**E**) ATMH, and (**F**) the loaded XG/PF-127 hydrogel.

The nature of new bond formations and changes in the chemical structure of developed hydrogels were elucidated by FTIR analysis. The FTIR spectra of all components are indicated in Figure 1. XG indicated FTIR spectra by bands at 3280 and 1608 cm^−1^, demonstrating hydrogen-bonded OH groups and COO– groups, whereas the bending of O–H and C–H was seen by peaks at 1018 and 1420 cm^−1^ [12,13], respectively. The FTIR spectra of PF-127 represent a C–O–C stretching vibration within the 1202–1002 cm^−1^ range. Likewise, the stretching vibration of COC and COC–CH_2_ was seen by peaks at 1098 and 1059 cm^−1^, whereas the CC–COC bond was confirmed by a peak at 1148 cm^−1^. Two characteristic bands were observed at 1342 and 1468 cm^−1^, indicating a CH_2_ group. An absorption peak at 2918 cm^−1^ was assigned to the methyl group [14,15]. Similarly, the FTIR spectrum of Aa indicated a stretching vibration of –CH_2_ by a broad band at 3014 cm^−1^. Furthermore, the stretching vibration of C=O of the carboxylic acid was presented by a peak at 1702 cm^−1^ [16]. The FTIR spectra of the XG/PF127 hydrogel indicated prominent peaks of XG, PF127, and Aa. Certain new peaks were formed while some were modified, indicating the successful crosslinking among hydrogel components. The prominent bands of XG and PF127 were changed from 3280, 1608 cm^−1^, and 2918, 1468 cm^−1^ to 2910, 1490, 2950, and 1505 cm^−1^, respectively. Similarly, the position of certain peaks of Aa were also modified, like bands at 3014 and 1702 cm^−1^ which were moved to 3042 and 1685 cm^−1^. This all demonstrates the synthesis of hydrogels. The FTIR spectrum of ATMH indicated the stretching vibration of the amino group (N–H) by a peak at 3270 cm^−1^, while the stretching vibrations of CH_2_ and COOH groups were observed at 2940 and 3322 cm^−1^, respectively. The C=C ring stretching and the C–H bending of CH_2_ groups were detected at 1598 and 1438 cm^−1^ [17]. After loading, a slight change was observed in the peaks of ATMH as 1438 and 2918 cm^−1^ were slightly shifted to 1412 and 2950 cm^−1^, representing the successful loading of ATMH by the XG/PF-127 hydrogel [18].

### 2.2. TGA

**Figure 2:**
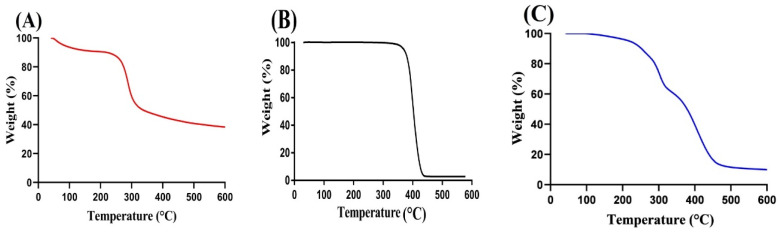
TGA of (**A**) XG, (**B**) PF-127, and (**C**) XG/PF-127 hydrogel.

TGA was performed for XG, PF-127, and prepared hydrogels as illustrated in Figure 2. The TGA curve of XG indicated a weight loss of 8 and 42% at 218 and 400 °C, respectively. The initial loss of weight was correlated with the release of volatile matter and absorbed water. On the other hand, stability can be seen by the TGA of PF-127 till 378 °C. Further rise in temperature resulted in the decomposition of PF127. A 95% weight loss was observed as temperature reached 423 °C. Increasing temperature led to a further decomposition of PF127, which still continued to entire degradation. The loss in the weight of PF-127 was due to the elimination of functional groups with the increase in temperature [19,20]. A degradation of 37% in weight was observed by TGA of XG/PF-127 as temperature approached 310 °C. This might be due to the removal of absorbed and bonded moisture and loss of hydroxyl groups. Similarly, a weight loss of 48% was seen at 487 °C. Further decomposition of developed hydrogel was seen with the increase in temperature. Comparing the thermal stability of XG and PF127 with the formulated hydrogel, we can demonstrate that the prepared hydrogel exhibited higher thermal stability than pure polymers, which basically indicated enhancement in the thermal stability of excipients after crosslinking among them [21].

### 2.3. DSC

**Figure 3:**
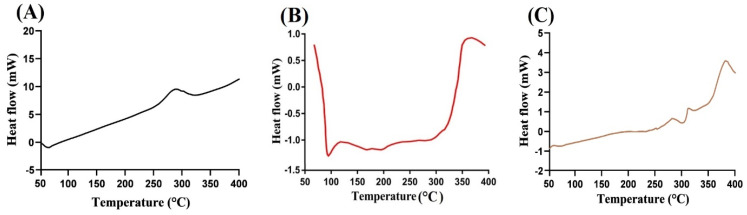
DSC of (**A**) XG, (**B**) PF-127, and (**C**) XG/PF-127 hydrogel.

The DSC curve of individual excipients and prepared hydrogel is shown in Figure 3. An exothermic peak was demonstrated at 65 °C while a broad endothermic peak was detected at 280 °C by XG’s DSC. The endothermic and exothermic peaks of XG indicated moisture loss and thermal decomposition. The DSC of PF-127 exhibited a strong endothermic peak at 70 °C, representing the devastation of crystalline network of the PF-127 chain. Similarly, an exothermic peak was seen at 113 °C. The developed hydrogel and individual components are quite compatible, as seen by the prepared formulation’s peaks migrating towards a higher glass transition temperature than the parent components [22] because of the higher intermolecular hydrogen bonding [23]. Therefore, greater thermal stability was indicated by the formulated hydrogel [24].

### 2.4. XRD Analysis

**Figure 4:**
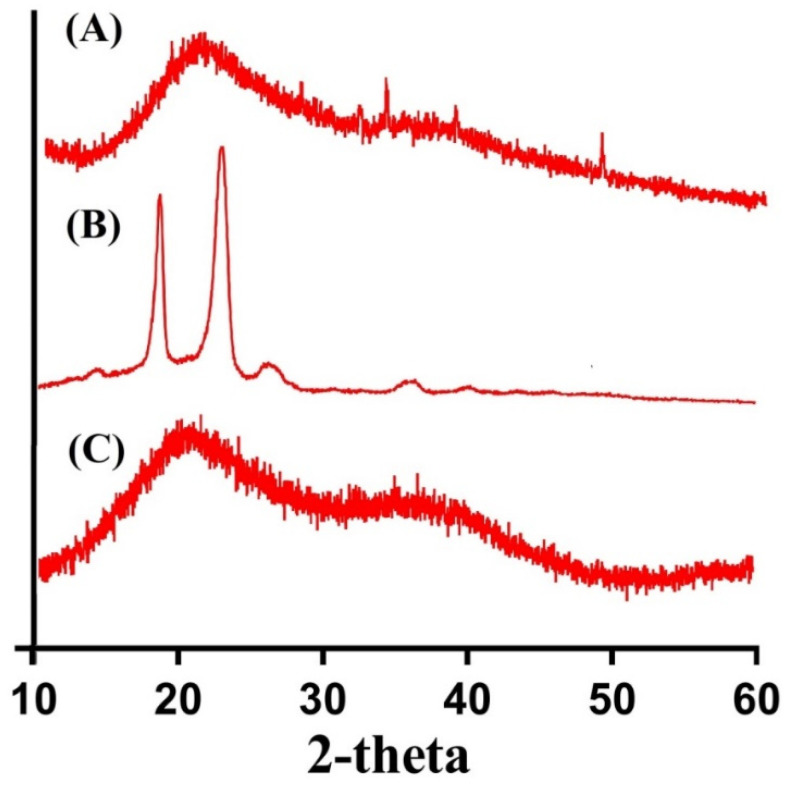
XRD of (**A**) XG, (**B**) PF-127, and (**C**) XG/PF-127 hydrogel.

The recorded XRD spectra of XG, PF127, and the formulated hydrogels are indicated in Figure 4. Prominent peaks were demonstrated by the XRD spectra of XG at 2θ = 20.19°, 32.10°, and 48.40°. XG is amorphous by nature and forms aggregates with the other side chains, hence restricting the proper packaging of polymer chains. Similarly, high sharp peaks of PF-127 were seen at 2θ = 19.20°, 21.83°, and 27.10°, respectively. The sharp high intense peaks of the PF-127 basically indicated its high stability and crystallinity. However, sharp and prominent peaks of XG and PF127 were replaced by dense peaks after crosslinking and polymerization reaction as indicated by the XRD analysis of the prepared hydrogel. All these factors indicate a decrease in pure reagent’s crystallinity after polymerization. The reduction in crystallinity of the reagents as indicated by polymeric hydrogel may be the feature of the formation of the conjugate of Aa with XG and PF127 in the presence of MBA, hence indicating enhancement in the fraction of the amorphous phase [25,26].

### 2.5. SEM

**Figure 5:**
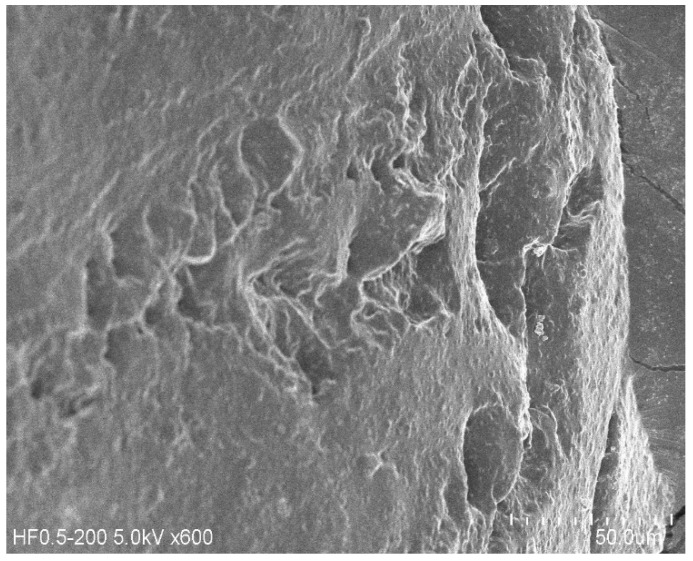
SEM of XG/PF-127 hydrogel.

The SEM of the XG/PF-127 hydrogel is illustrated in Figure 5. The prepared network displayed a hard surface with a few big pores, which may be connected to high crosslinking of the XG and PF-127 with the Aa content. The strong crosslinking among hydrogel reagents improved the mechanical strength and stability of the hydrogel; thus, it can be used as a controlled drug delivery carrier [27].

### 2.6. Sol–Gel Analysis

The cross-linked and uncross-linked fractions of the synthesized hydrogel were estimated by sol–gel analysis (Table 1). Gel is the cross-linked while sol is the uncross-linked fraction of the formulated hydrogel. Both sol and gel fractions were influenced highly by the incorporated reagents of hydrogel. With an increase in the XG and PF127 feed ratios, the gel fraction rose. During polymerization reaction, free radicals are produced, which leads to the crosslinking of XG and PF-127 with Aa by MBA. Thus, as the feed ratios of XG and PF127 increase, more free radicals are generated in the same way. Thus, greater reactive sites are available for the monomer to crosslink with the polymers. Similarly, high incorporated feed ratios of Aa also resulted in high levels of gel formation. Aa plays a key role in polymerization of hydrogel reagents. Crosslinking density of the hydrogel is improved with the high incorporated contents of Aa, thus high gel fraction is achieved. In other words, we can say that like XG and PF127, high feed rations of Aa also result in high gel fraction [27]. Khalid and coworkers reported high gel fraction with the high feed ratios of hydrogel reagents [28]. Unlikely, a reduction was observed in the sol fraction with the high incorporated hydrogel contents [29]. Nasir et al. (2019) reported low sol with high gel fractions for the developed gels with their high incorporated contents [30].

### 2.7. Porosity

**Figure 6:**
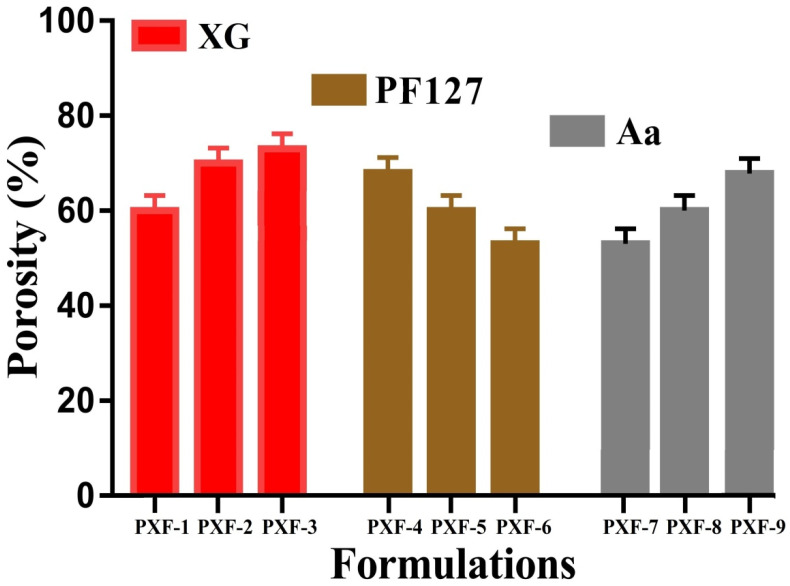
Effect of XG, PF-127, and Aa on porosity of XG/PF-127 hydrogel.

The swelling and drug loading of the hydrogel and its sub-micro/nano particulate systems are dependent completely on their porosity. High porosity resulted in maximum swelling and loading of the drug. Porosity study was performed for the hydrogel formulations. Porosity was affected by different incorporated feed ratios of the hydrogel contents (Figure 6). With XG’s increased feed ratios, a rise in porosity was seen. Similarly, an increase was seen in porosity with the high feed ratios of Aa. This may be correlated with the formation of highly viscous mixture during the polymerization process, which restricted the evaporation of bubbles; as a result, interconnected channels were produced. Water molecules penetrated into the hydrogel networks through these channels, and thus high porosity was achieved. Unlike in the case with XG and Aa, PF127 also affected the porosity, but in a reverse way. An increase in PF127 contents resulted in a low porosity due to the formation of a highly cross-linked network, which increased the hardness and decreased the pore size of the prepared network. Hence, it can be demonstrated that an increase in feed rations of PF127 and a drop in porosity are observed while an increase in porosity is seen with the high incorporated feed ratios of XG and Aa [31,32].

### 2.8. Swelling Study

**Figure 7:**
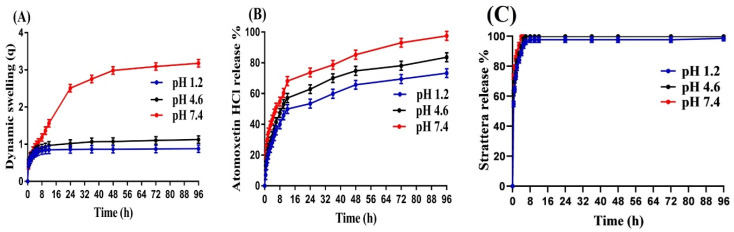
Effect of pH on (**A**) swelling, (**B**) drug release from XG/PF-127 hydrogel, and (**C**) commercial product Strattera.

The swelling degree of hydrogels is shown in Figure 7A, indicating a low swelling index at pH 1.2 and 4.6; however, it augmented considerably with the increase in pH of the medium, i.e., to pH 7.4. This change in swollen hydrogels due to pH changes can occur due to the development of osmotic swelling forces. These forces are generated by the carboxyl groups (Aa) present in the hydrogel network. Carboxyl groups begin to ionize at pH 4 and completely ionize above pH 6. The existence of more ionic groups in the hydrogel network at high pH resulted in maximum swelling. The prepared XG/PF-127 hydrogel consist of both COO^−^ and –COOH groups. These groups can change into one another under favorable conditions. At pH 1.2, –COO groups protonated into –COOH, but at basic pH 7.4, –COOH entirely deprotonated back into –COO groups. Moreover, XG contains *O*-acetyl, pyruvyl and unreacted hydroxyl groups, which also undergo deprotonation at pH > 6. Thus, high charge density was produced, and thus high electrostatic repulsion between negatively charged –COO^−^ groups occurred, which led to high swelling of prepared hydrogel at higher pH values [33]. The hydrogen-bonding strength among hydrogel networks is most likely to be strengthened by the large ratio of protonated COOH groups, and thus low swelling was observed at pH 1.2 and 4.6 [30]. Similarly, hydrogel contents also have a significant impact on the hydrogel’s swelling. A rise in swelling was seen with the high feed ratios of XG and Aa because of their high functional groups, while in case of PF-127, a drop was observed in swelling with the high levels of the incorporated PF-127 contents. The reason may be attributed to the formation of a hard and bulk network of hydrogel which did not allow the sufficient water molecules to enter into the hydrogel networks [34,35].

### 2.9. Drug Loading and In Vitro Drug Release Studies

Drug loading was conducted for XG/PF-127 hydrogels as illustrated in Table 2. Like porosity and swelling, the contents of the hydrogel also affected the drug loading. Greater drug loading was detected with the high feed ratios of XG and Aa. The possible reason is the high porosity and swelling index of the hydrogel which occurred with the increase in XG and Aa contents [36,37]. Contrary to XG and Aa, a drop was seen in drug loading by the synthesized matrix as the feed ratios of PF-127 were enhanced [38].

Drug release tests were carried out at pH 1.2, 4.6, and 7.4 for the prepared hydrogels and commercial product Strattera (Figure 7B,C). Maximum release of the drug was seen at high pH values due to the deprotonation of XG and Aa functional groups, whereas minimum drug release was detected at pH 1.2 due to the protonation of such functional groups [39,40]. The release studies of the Strattera indicated a rapid release of drug at all three pH values. Almost a 90% concentration of the drug was released at pH 7.4 within the initial 2 h, while in the case of pH 4.6 and 1.2, a concentration of more than 80% of the drug was released within the initial 2 h. Comparing the drug release of Strattera and that of the prepared hydrogel, we can predict that the drug was sustained successfully for extended period of time by the developed hydrogels.

Similar to swelling, the hydrogel’s contents also have an impact on drug release. High drug release was observed with the high incorporated XG and Aa contents [41], while a decline was perceived in drug release with the high PF-127 contents [42] and vice versa.

### 2.10. Release Mechanism

To establish the sequence and drug release from the prepared networks, multiple kinetic models were computed using the release data of the synthesized hydrogels. “r” values determine the most suitable kinetic order. Table 2 indicates that the “r” values for the first order were higher than the “r” values of all other kinetic models. As a result, we can state that all formulations of the prepared hydrogel follow the first order of kinetics. The diffusion type is determined by the “*n*” value. If 0.45 > *n*, Fickian diffusion occurs; otherwise, non-Fickian or anomalous transport similar to linked diffusion/polymer relaxation occurs. [43]. All formulations have “*n*” values between 0.5040 and 0.5790, indicating non-Fickian diffusion [44].

## 3. Conclusions

The free radical polymerization technique was adopted for the development of XG/PF-127 hydrogels. A stimuli-responsive effect was manifested by the graft copolymer. The controlled release of the ATMH was detected at pH 7.4 for 96 h. The compatibility among the various formulation components of the developed hydrogel was shown by FTIR. DSC and TGA showed an increase in the thermal stability of XG and PF-127 after the polymerization process. Similarly, SEM indicated a hard surface with few large pores through which water penetration occurred. After a polymerization process, XRD showed a reduction in the crystallinity of polymers. High swelling index was seen at pH 7.4, whereas at pH 1.2 and 4.6, low swelling was observed. Hence, it could be inferred from the results that the XG/PF-127 hydrogel can be employed as a potential agent for controlled drug delivery, which would be helpful not only in minimizing undesired GIT effects, but also in enhancing patient compliance and therapeutic output.

## 4. Materials and Methods

### 4.1. Materials

ATMH was obtained from Hetero labs limited (UNITE I), Telangana, India. Similarly, Xanthan gum was purchased from Tokyo Chemical Industry Co., Ltd., (Tokyo, Japan). Acrylic acid was purchased from Acros (Carlsbad, CA, USA). Likewise, pluronic F127 and ammonium persulfate were obtained from Sigma-Aldrich (Chemie GmbH, Riedstir-Steinheim, USA) and Showa (Tokyo, Japan). N,N′-Methylene bisacrylamide (MBA) was acquired from Alfa Aesar (Lancashire, UK), respectively.

### 4.2. Methods of Preparation

Xanthan gum (XG)- and pluronic (PF-127) [XG/PF-127]- based hydrogels were prepared by the free radical polymerization technique. A pre-weighed quantity of XG was dissolved in distilled water at 40 °C for 6 h. The PF-127 solution was mixed with the XG solution under constant stirring. Ammonium persulfate (APS) was poured into the mixture. After that, acrylic acid (Aa) was added in to the XG, PF127 and APS mixture. Finally, an MBA solution was mixed with the aforementioned mixture to crosslink the polymers and monomer on their specific sites. The entire mixture was kept on stirring until a transparent solution was formed, purged by nitrogen gas and then transferred into the glass molds, which were positioned in the water bath. The temperature of the water bath was maintained at 55 °C initially for 2 h, which was increased up to 65 °C later. The formulated gel was cut into 8 mm discs. A mixture of distilled water and ethanol was used for washing the prepared discs of gels. After that, hydrogel discs were placed for one week in a vacuum oven for dehydration. The dried discs were processed further for different studies. A set of formulations is shown in Table 3.

### 4.3. Characterization

FTIR, TGA, DSC, XRD, and SEM were performed according to our previous publication [45].

### 4.4. Sol–Gel Anaylsis

The sol and gel contents of the synthesized hydrogel were estimated by sol–gel analysis. The unreacted part of the hydrogel is known as the sol fraction, while the reactant part is known as the gel fraction. The weighed discs of the prepared hydrogel were placed for 12 h in distilled water. After that, the discs were removed and positioned in the oven for dryness. The dehydrated discs were weighed again [46]. Equations (1) and (2) were applied for the estimation of sol and gel fractions.

(1) $$\mathrm{Sol}\;\mathrm{fraction}\%=\frac{{\;C}_{1}-{\;C}_{2}}{{\;C}_{1}}\times 100,$$

(2) Gel fraction=100−Sol fraction.

Here, C_1_ represents the initial weight of dried hydrogel before the extraction process, while C_2_ is the final weight after the extraction process.

### 4.5. Porosity

The porosity study of the prepared hydrogels was performed in absolute ethanol. Hydrogel discs were taken, weighed (A_1_) and immersed in ethanol for 3 days. After achieving equilibrium swelling, discs were removed and weighed (A_2_) again [47]. Equation (3) was employed for the determination of porosity of the prepared hydrogels.

(3) $$(\%)\;\mathrm{Porosity}=\frac{A_{2}-A_{1}}{\rho V}\times 100.$$

Here, ρ is the density of absolute ethanol while V is the swelling volume of hydrogel discs.

### 4.6. Swelling Study

Weighed hydrogel discs were soaked in buffer solutions of pH 1.2, 4.6 and 7.4. The discs were removed at a specific interval of time, blotted with filter paper, weighed, and placed again in the respective media [48]. Dynamic swelling (q) was determined by Equation (4).

(4) $$\left(q\right)=\frac{{\;T}_{2}}{T_{1}},$$

where T_2_ indicates the weight of the swollen hydrogel disc and T_1_ represents the weight of the dried hydrogel disc at time t.

### 4.7. Drug Loading

A pH 7.4 phosphate buffer solution was used to prepare a 1% drug solution. For four days, precisely weighed hydrogel discs were submerged in the drug solution. Following that, discs were removed and cleaned with distilled water to remove any drugs that adhered to the hydrogel discs’ surface. The cleaned discs were placed in a vacuum oven to dry [49].

### 4.8. In Vitro Drug Release Study

USP dissolution apparatus-II was employed for the drug release study. The release of the drug from the formulated hydrogel and commercial product Strattera (60 mg) (ELI LILLY and Co., Ltd.) was investigated at three different pH values, i.e., pH 1.2, 4.6 and pH 7.4. XG/PF127-loaded hydrogel discs and the commercial product were placed in 900 mL of each buffer solution with 50 rpm at 37 ± 0.5 °C. Samples of 5 mL were collected and fresh medium of the same quantity was added back to maintain constant sink conditions. The collected samples were analyzed by using a UV spectrophotometer (U-5100, 3 J2-0014, Tokyo, Japan) at the wavelength (λmax) of 226 nm [50].

### 4.9. Release Mechanism

The order and release mechanism of the drug from the prepared hydrogel was determined by using various kinetic models. The release data were fitted into zero-order, first-order, Higuchi, and Korsmeyer–Peppas models [51], respectively.

### 4.10. Statistical Analysis

SPSS Statistic software 22.0 (IBM Corp, Armonk, NY, USA) was performed for the statistical analysis of all experimental data. Differences between the tests were determined by Student’s t-Test and considered significant statistically (*p*-value < 0.05).

## Figures and Tables

**Table 1:** Drug loading and polymer volume fraction of XG/PF-127 hydrogels.

Formulation Code	Sol Fraction (%)	Gel Fraction (%)	Drug Loaded (mg)/350 mg of Dry Gel	
			Weight Method	Extraction Method
PXF-1	11	89	132.10 ± 0.71	130.84 ± 1.03
PXF-2	9	91	155.23 ± 0.93	152.42 ± 0.64
PXF-3	7	93	164.01 ± 1.10	163.02 ± 0.85
PXF-4	15	85	102.34 ± 0.78	101.04 ± 0.94
PXF-5	12	88	93.61 ± 0.93	91.23 ± 1.01
PXF-6	10	90	86.03 ± 1.03	84.87 ± 0.84
PXF-7	16	84	143.44 ± 0.87	142.31 ± 0.92
PXF-8	14	86	161.05 ± 0.91	159.92 ± 0.48
PXF-9	13	87	170.72 ± 0.83	168.63 ± 1.20

**Table 2:** Kinetic modeling release of drug from XG/PF-127 hydrogels.

F. Code	Zero Order	First Order	Higuchi	Korsmeyer–Peppas	
	r^2^	r^2^	r^2^	r^2^	N
PXF-1	0.9432	0.9920	0.8937	0.9582	0.5331
PXF-2	0.9356	0.9978	0.9220	0.9819	0.5562
PXF-3	0.9682	0.9870	0.9712	0.9754	0.5040
PXF-4	0.9548	0.9764	0.9692	0.9625	0.5128
PXF-5	0.9793	0.9954	0.9830	0.9922	0.5673
PXF-6	0.9450	0.9788	0.9706	0.9414	0.5468
PXF-7	0.9890	0.9903	0.9790	0.9378	0.5219
PXF-8	0.9063	0.9661	0.9332	0.9627	0.5493
PXF-9	0.9274	0.9845	0.9568	0.9439	0.5790

**Table 3:** Feed ratio scheme for formulation of XG/PF-127 hydrogels.

F. Code	Polymer (XG) g/30 g	Polymer (PF-127) g/30 g	Monomer (Aa) g/30 g	Initiator (APS) g/30 g	Cross-linker (MBA) g/30 g
PXF-1	0.080	0.200	4.0	0.1	0.2
PXF-2	0.120	0.200	4.0	0.1	0.2
PXF-3	0.160	0.200	4.0	0.1	0.2
PXF-4	0.050	0.300	4.0	0.1	0.2
PXF-5	0.050	0.350	4.0	0.1	0.2
PXF-6	0.050	0.400	4.0	0.1	0.2
PXF-7	0.050	0.200	4.5	0.1	0.2
PXF-8	0.050	0.200	5.0	0.1	0.2
PXF-9	0.050	0.200	5.5	0.1	0.2
